# Radiation Effects in Electret Organic Thin‐Film Transistors Due to High Flux and High Dose X‐Ray Irradiation

**DOI:** 10.1002/adma.202508402

**Published:** 2025-11-29

**Authors:** Alexandria Mitchell, Jessie A. Posar, James Cayley, Georgia York, Michael Lerch, Attila J. Mozer, Igor Píš, Elena Magnano, Luca Tosti, Maddalena Pedio, Alasdair Syme, Ian G. Hill, Marco Petasecca

**Affiliations:** ^1^ Department of Physics & Atmospheric Science Dalhousie University Halifax NS B3H 4R2 Canada; ^2^ School of Physics University of Wollongong Wollongong NSW 2522 Australia; ^3^ Intelligent Polymer Research Institute University of Wollongong Wollongong NSW 2522 Australia; ^4^ CNR ‐ Istituto Officina dei Materiali (IOM) Basovizza Trieste 34149 Italy; ^5^ Sezione Perugia CNR ‐ Istituto Officina dei Materiali (IOM) Via Pascoli s.n.c. Perugia 06123 Italy; ^6^ Sezione di Perugia Istituto Nazionale di Fisica Nucleare Via Pascoli s.n.c. Perugia 06123 Italy; ^7^ Department of Medical Physics Nova Scotia Health Authority Halifax NS B3H 1V7 Canada

**Keywords:** organic semiconductors, radiation dosimetry, synchrotron radiation, x‐ray photoelectron spectroscopy

## Abstract

Organic electronic devices offer lightweight, flexible, and low‐cost alternatives to conventional semiconductor technologies, with growing interest in dosimetry applications. An organic thin‐film transistor (OTFT) with a polymer electret is presented for high dose‐rate synchrotron dosimetry. The OTFT operates in accumulated‐dose and real‐time readout modes and demonstrates excellent linearity with various beam filtrations. Device response increases with decreasing energy, and simulations reveal gold contacts as the primary source of energy dependence. Depth dose measurements show good agreement with a commercial detector, validating dosimetric performance. Minimal changes in mobility are observed at clinically relevant doses, but mobility degradation becomes apparent after high accumulated doses, indicating radiation damage in active materials. X‐ray photoelectron spectroscopy (XPS) and near‐edge X‐ray absorption fine structure (NEXAFS) techniques are employed to analyze pristine and irradiated active material films separately and in a combined stack. XPS reveals oxidation in pentacene and a Fermi level shift in polystyrene in irradiated films, both of which likely cause the mobility reduction observed in OTFTs. Valence band and NEXAFS spectra show no evidence of new states in the bandgap. These findings demonstrate the potential of OTFTs as dosimeters for high dose‐rates and clarify how radiation alters the molecular structure and electronic behavior of the device.

## Graphical abstract

Electret‐style organic thin‐film transistors are evaluated for synchrotron X‐ray dosimetry at ultra‐high dose rates, showing promising performance and agreement with standard detectors. XPS and NEXAFS reveal radiation‐induced oxidation and Fermi level shifts in pentacene and polystyrene, correlating with reduced charge carrier mobility and providing insight into radiation degradation mechanisms in organic semiconductors.

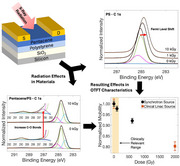

## 1 Introduction

Organic semiconductors are a class of materials of increasing interest for many different applications, such as organic light‐emitting diodes,^[^ ^1^ ^]^ solar cells,^[^ ^2^, ^3^ ^]^ biosensors,^[^ ^4^ ^]^ and radiation detection.^[^ ^5^, ^6^, ^7^ ^]^ Organic materials allow for the fabrication of low‐cost, flexible, electronic devices with the capability of large‐scale arrays.^[^ ^8^ ^]^ Organic electronics are an attractive candidate for radiation dosimetry applications due to their inherently low atomic number (Z) and density of the materials. Soft tissue has an effective Z of ≈7.4, while organic materials are mainly comprised of carbon (Z = 6), offering a comparable composition. These properties allow for the possibility of fabricating a tissue equivalent dosimeter – a device that mimics the response of the human body to radiation. High Z materials respond differently to radiation than tissue, leading to strong variations of their response to lower energy photon irradiation, in particular.^[^ ^9^ ^]^ Silicon (Z = 14) is a material used for diodes and Metal‐Oxide‐Field‐Effect‐Transistors and often adopted to manufacture electronic dosimeters. They have shown to have up to ≈7‐fold overresponse at low‐energy x‐rays (20–100 keV) compared to 6 MV photon beams.^[^ ^10^, ^11^ ^]^ Organic electronic devices in the form of photoconductors,^[^ ^12^ ^]^ photoresistors,^[^ ^13^ ^]^ diodes,^[^ ^14^, ^15^, ^16^, ^17^ ^]^ and transistors^[^ ^18^, ^19^ ^]^ have shown promising responses to radiation and have reported energy independence over a wide energy range.^[^ ^20^ ^]^

Organic thin film transistors (OTFTs) have demonstrated suitability as radiation dosimeters through measurable changes in key electrical parameters, including threshold voltage shift, charge carrier mobility, and on/off currents. The off current of OTFTs has been shown to have a proportional increase with dose, poly 3‐hexylthiophene (P3HT) and pentacene‐based OTFTs have demonstrated off current sensitivity of 4.4 and 26.7 nA Gy^−1^ respectively, for 50 Gy with a Cobalt‐60 (Co‐60) source.^[^ ^21^ ^]^ For pentacene OTFTs with SiO_2_ gate dielectric, mobility has been reported to degrade by 80% after 1200 Gy with Co‐60 source, but this is reduced to a 30% degradation with a polyimide dielectric.^[^ ^22^ ^]^ OTFTs using 2,8‐difluoro‐5,11‐bis(triethylsilylethynyl)‐anthradithiophene (diF‐TES‐ADT) have demonstrated a negative voltage shift with a sensitivity of 0.46 ± 0.09 V Gy^−1^ from 1 to 10 Gy in response to 6 MV x‐rays.^[^ ^23^ ^]^ A blend of 6,13‐Bis(triisopropylsilylethynyl) (TIPS)‐pentacene and polystyrene (PS) used as an organic semiconductor in an OTFT demonstrated a negative voltage shift with dose and a very high sensitivity of 2.8 V Gy^−1^ up to 3 Gy with Co‐60 gamma rays.^[^ ^24^ ^]^

**Figure 1:**
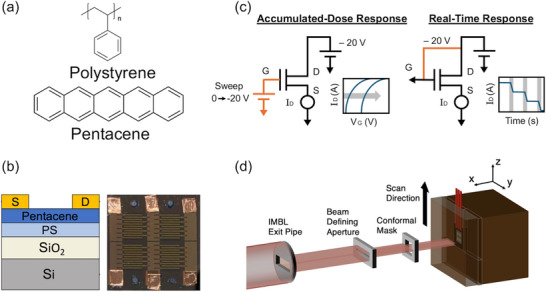
a) Chemical structure of the organic materials used in the organic thin film transistors (OTFTs), polystyrene (PS) and pentacene. b) Schematic of the structure of the stacked materials in the OTFTs and an optical image of the top view of the pixelated device. c) The source measure unit (SMU) connections and voltages used for the two different readout methods possible for the electret OTFTs to monitor dose. d) Diagram of the irradiation setup at the Imaging and Medical Beamline (IMBL) of the device in a solid water phantom and the synchrotron beam scanning across the face of the device.

Among the various organic semiconductors used in electronic devices, pentacene is one of the most widely studied. It is a small molecule semiconductor that has reported high mobilities (> 1 cm^2^ V^−1^ s^−1^) in OTFTs.^[^ ^25^ ^]^ In addition to its electrical performance, it also has a low density (1.3 g cm^−3^) that is similar to that of water or soft tissue (≈1.0 g cm^−3^) and is just comprised of five linearly fused benzene rings (**Figure** **1**a). Our group has developed a pentacene OTFT with a polymer electret layer to be used for radiation dosimetry applications. An electret is a dielectric material that can retain a quasi‐permanent electric field in the absence of an applied external field.^[^ ^26^, ^27^ ^]^ The electric field is created by briefly applying a high programming voltage to the gate, which traps charges in the electret layer and temporarily shifts the threshold voltage. PS is the polymer used for the electret layer in this OTFT, due to its good charge retention and large memory window in polymeric memory devices.^[^ ^26^, ^28^ ^]^ Additionally, it is comprised of only hydrogen and carbon (Figure 1a), has a low density of ≈1 g cm^−3^, and is solution‐processable. The addition of the electret allows for these OTFTs to measure dose without applying an external bias during irradiation. Thus, developing the possibility for a compact, wearable, and wireless radiation dosimeter for practical use in clinical settings. Preliminary characterization of these electret OTFT devices in clinically relevant settings with MeV and keV photon beams was presented in our previous work, where they demonstrated excellent linearity with dose.^[^ ^29^ ^]^ They have also been fabricated on flexible plastic substrates and have shown the capability of real‐time detection.^[^ ^30^ ^]^

The use of clinical linear accelerators and orthovoltage x‐ray tubes has limited the studies available in the literature to electrical characterisations based on relatively low dose rate exposures. There is a growing interest in ultra‐high dose rate (>40 Gy s^−1^) radiation. Such high dose rates have been adopted in advanced clinical applications because they have shown an increasing differential response between normal and tumour tissue, referred to as the FLASH effect.^[^ ^31^ ^]^ Preclinical studies have verified that FLASH can reduce normal tissue damage while maintaining tumour control,^[^ ^32^, ^33^ ^]^ and when the first patient trial was conducted, demonstrated a satisfactory clinical outcome with respect to normal tissue and tumour response.^[^ ^33^ ^]^ This study aims to evaluate the response of these OTFTs in ultra‐high dose rate environments using synchrotron x‐rays, an area of research that is currently absent from the literature.

Along with characterizing OTFT's response to synchrotron radiation, it is also of interest to investigate the underlying mechanisms of radiation response in the organic materials adopted. While the radiation response of typical inorganic semiconductors (silicon, germanium) is well documented,^[^ ^34^, ^35^, ^36^ ^]^ their organic counterparts remain less understood. Some studies have linked performance changes to radiation‐induced material damage, such as C─C and C─H bond scission.^[^ ^37^, ^38^ ^]^ Other work has emphasized the role of charge trapping and de‐trapping processes in the organic semiconductor and dielectric.^[^ ^24^, ^39^ ^]^ Dremann et al. have recently shown that radiation dose increases electronic trap density in diF‐TES‐ADT OTFTs, and these traps originated from local structural disorder within the crystalline films.^[^ ^40^ ^]^ To investigate the radiation‐induced changes in the organic materials used in our electret‐style OTFTs, this study employs the use of X‐ray Photoelectron Spectroscopy (XPS) and Near Edge X‐ray Absorption Fine Structure (NEXAFS) spectroscopy techniques. XPS has been employed to probe the elemental composition, oxidation state and functional groups present.^[^ ^41^ ^]^ While XPS probes the occupied electronic states, NEXAFS provides element‐specific information about the local electronic structure, utilizing the strict dipole selection rule for electronic transitions from deep core levels to the unoccupied orbital states. In particular, the changes in energy position and intensity of C K‐edge NEXAFS resonances can be used to study changes in molecular bonding upon chemical or structural modifications.^[^ ^42^ ^]^ Therefore, both these spectroscopy techniques were used to gain insight into chemical and electronic structure modifications in the organic materials used in the OTFTs after high radiation doses. Specifically, looking for defects, traps, and emission centers in the bandgap which can be related to changes seen in OTFT characteristics with dose.

## 2 Results and Discussion

### 2.1 Detection Performance of Electret‐Style OTFTs with Synchrotron X‐rays

OTFTs were fabricated in a top contact/bottom gate configuration on heavily doped (n‐type) Si substrates with 300 nm of thermally grown SiO_2_ to act as the gate and gate dielectric, respectively, for the transistor. These OTFT devices consist of a polymer electret layer (45 nm PS), organic semiconductor (50 nm pentacene), and gold interdigitated electrodes (50 nm) (Figure 1b). The device fabrication process has been described in our previous work.^[^ ^29^ ^]^ Each substrate was 25 × 25 mm^2^ and consisted of 4 individual transistors.

The current–voltage (*I*–*V*) characteristics of the OTFTs were measured with a dual‐channel Source Measure Unit (SMU) (Keithley 2614B). Errors associated with *I*–*V* measurements with this specific SMU are assumed to be 0.02% + 5 mV for voltage and 0.025% + 500 pA for current readings, respectively.^[^ ^43^ ^]^ The OTFTs were used to measure dose in two readout modes, accumulated‐dose and real‐time. Connections for the two readout methods are shown in Figure 1c. In accumulated‐dose mode, a high bias of −80 V was applied to the gate for 3 s before irradiation to program the electret OTFT and temporarily shift the *I*–*V* characteristics, shifting the threshold voltage negatively. This programming bias is trapping charges in the polymer electret layer of the OTFT, likely through the injection of holes from the pentacene organic semiconductor into the PS electret.^[^ ^26^, ^44^, ^45^ ^]^ OTFTs without an electret layer do not exhibit a considerable shift after a programming bias, suggesting that the traps are primarily being created in the polymer dielectric.^[^ ^26^, ^44^ ^]^ The OTFT was then read out immediately before and after irradiation with no bias applied during the irradiation. Variation in *I*–*V* curves pre‐ and post‐irradiation were used to calculate the dose. During real‐time readout, a bias was applied during irradiation, and the change in current due to irradiation was monitored in real‐time. The current was then integrated to determine the collected charge during irradiation.

The OTFT's dose response was characterized under high‐dose‐rate synchrotron x‐rays with the Imaging and Medical Beamline (IMBL) at the Australian Synchrotron.^[^ ^46^, ^47^, ^48^ ^]^ The energy and dose rate of the beam were altered with a combination of Molybdenum (Mo), Copper (Cu), and/or Aluminum (Al) filters placed in front of the beam. Reference dosimetry was performed using a PTW 31 022 PinPoint ionization chamber under reference conditions (20 mm depth, 20 × 20 mm^2^ field size) with a scan speed of 10 mm s^−1^. Beam characteristics are outlined in **Table** **1**. For dose measurements with the OTFT, it was placed at a depth of 20 mm in a water‐equivalent phantom (solid water), face‐on with the beam. The broad beam was scanned across the face of the OTFTs (z‐direction) to assess the response. A schematic of the beam and OTFT during irradiation is shown in Figure 1d. In the accumulated‐dose readout mode, the Mo‐Mo scans were conducted at a speed of 10 mm s^−1^ (exposure time of 2 s) and for Cu‐Cu/Al‐Cu scans were at a speed of 20 mm s^−1^ (exposure time of 1 s). In real‐time readout mode, measurements were taken for all filter conditions at 20 mm s^−1^.

**Table 1:** Reference dosimetry of the IMBL's 3T broad beam taken with a PTW 31 022 PinPoint ionization chamber at reference dosimetry conditions and at a scan speed of 10 mm s^−1^.

Beam filtration	Average energy [keV]^a)^	Delivered dose [Gy]	Dose rate [Gy s^−1^]
Mo‐Mo	124	3.121	29.43
Cu‐Cu	95.1	20.88	197.1
Al‐Cu	82.9	53.94	509.3

^a)^ Energies taken from Livingstone et al.^[^ ^49^ ^]^

**Figure 2:**
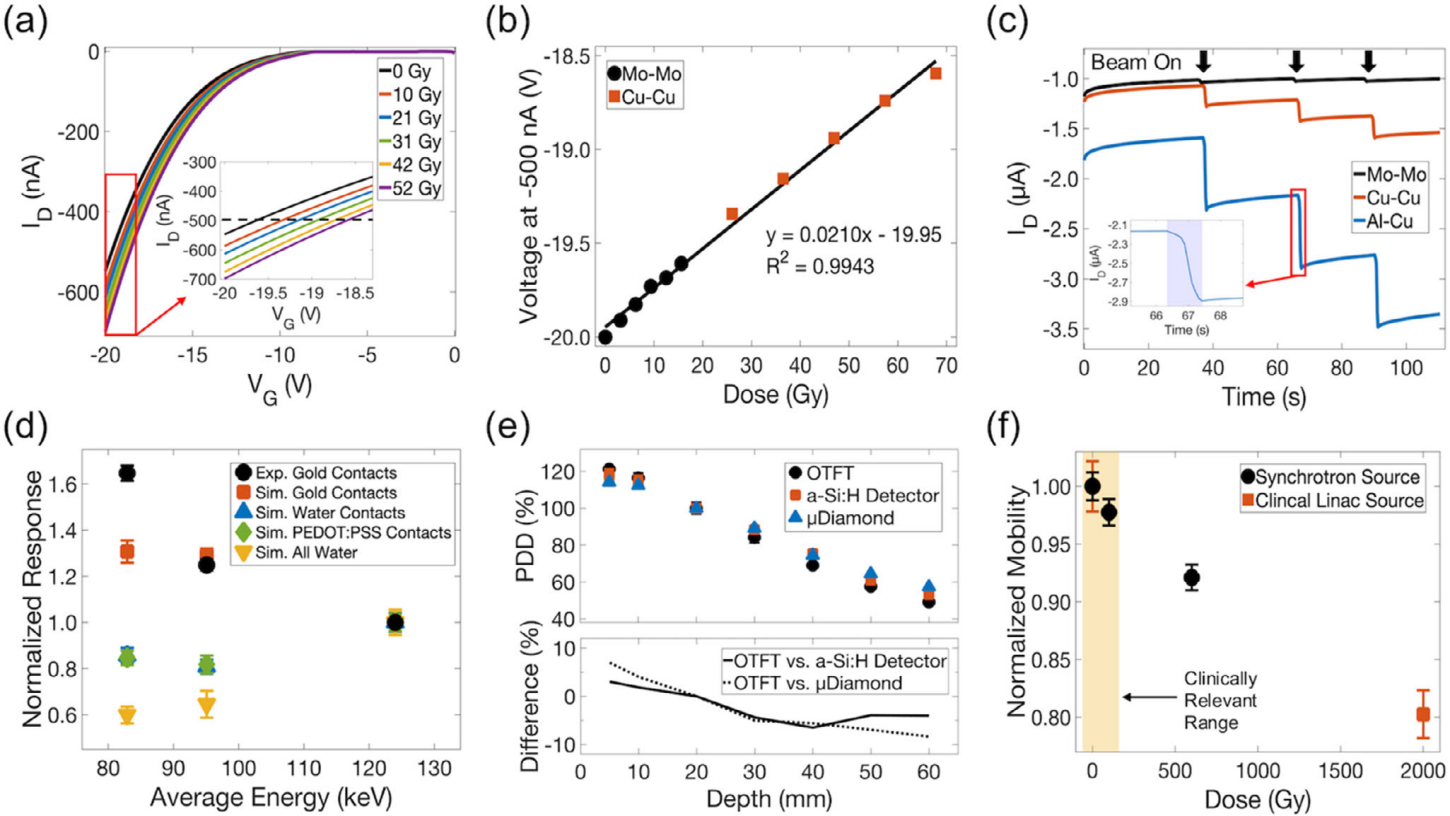
a) An example of the data collected in the accumulated‐dose readout mode of the OTFT with PS electret. *I*–*V* curves are measured at V_D_ = −20 V, 0 Gy curve is after applying programming bias of −80 V to the gate for 3 s and other curves were taken immediately after subsequent irradiations with Cu‐Cu filter. Doses represent the accumulated dose to the device. Inset demonstrates the separation between curves. b) Gate voltage at −500 nA taken from the *I*–*V* curves as a function of accumulated dose for irradiations with Mo‐Mo and Cu‐Cu filtrations. Error bars are smaller than markers, error is from measurement error of SMU. c) Real‐time readout of OTFT, V_D_ = V_G_ = −20 V and drain current monitored with time. Irradiation from Mo‐Mo, Cu‐Cu, and Al‐Cu filtrations for 3 repetitions each for statistics. Inset shows a closer view of the current change as the beam is on. d) Normalized response for each filter condition from real‐time readout shown as a function of average energy for each filter. Experimental data points represent the mean integral of the current when the beam is on from 3 repetitions and error bars represent the standard deviation. Simulation data points represent the simulated energy deposited in the pentacene layer of the device and error bars are the calculated error. e) Percent depth dose (PDD) for Mo‐Mo filtered beam measured with OTFT in real‐time readout. Comparisons with PTW microdiamond detector^[^ ^49^ ^]^ and a hydrogenated amorphous silicon (a‐Si:H) diode.^[^ ^56^ ^]^ Data points represent the mean of 3 repetitions and error bars are the standard deviation, which are smaller than the markers for most points. f) Normalized mobilities calculated from transfer curves as a function of accumulated dose. One device was irradiated at the AU synchrotron with beams of average energies 50–125 keV and the other was irradiated with a linear accelerator (linac) with a 6 MV FFF beam. Highlighted region demonstrates the range at which would be relevant for most clinical applications.

**Figure** **2**a presents the raw data collected with the accumulated‐dose response of the OTFT and the shift exhibited in the transfer characteristics with cumulative radiation dose; the inset shows a close‐up of the separation between the curves at higher voltages. After irradiation, the threshold voltage shifted positively, back toward the initial state before applying the programming bias. Our working theory for the observed shift with dose is that the holes trapped in the polymer electret from the negative programming bias are recombining with electrons from electron‐hole pairs generated by ionizing radiation and de‐trapping. Similar to the effect that applying a high positive bias to the gate would have, reversing the programming bias.^[^ ^45^ ^]^ However, this remains to be shown. To confirm that the PS electret layer is responsible for the programming effect and enables the OTFTs to respond to radiation, experiments were performed with identical devices without the PS layer and devices that were not programmed before irradiation. In both cases, no response due to radiation was observed, and OTFTs without PS exhibited completely different behavior (see Figure S1, Supporting Information for more details).

The gate voltage shift measured at a set current of −500 nA trends linearly with cumulative dose for the combined data set of Mo‐Mo and Cu‐Cu filtered irradiations (Figure 2b). The sensitivity of the OTFT was determined from the slope of the voltage vs dose relationship, and the uncertainty was calculated from the standard error of the linear fit, which was (21.0 ± 0.5) mV Gy^−1^ for Mo‐Mo and Cu‐Cu filter. With the same OTFT, linearity was also assessed for the Al‐Cu filter (Figure S2, Supporting Information); sensitivity was found to be (14.8 ± 0.3) mV Gy^−1^ for Mo‐Mo and Al‐Cu filters up to 70 Gy. OTFT response after 70 Gy approached saturation with a decreasing sensitivity. However, when the programming bias was applied again (trapping more charges), it reset the OTFT and recovered linearity. With repeated use of the same OTFT, the sensitivity changed in accumulated‐dose mode, conclusions cannot be drawn about the differences in sensitivity between filter conditions (i.e., energy) when used in this mode. Further investigations are needed on the stability of these electret OTFT devices to predict how sensitivity changes with repeated use and reprogramming, including monitoring temperature changes, as variations can induce annealing effects that may influence stability and sensitivity.^[^ ^50^ ^]^

From the *I*–*V* curves measured, the mobility (μ) and threshold voltage (V_T_) were calculated at each dose step, using the square root of the drain current (I_D_) as a function of gate voltage (V_G_). In an ideal field‐effect transistor, this relationship is linear above threshold in saturation transfer curves, and the V_T_ is extrapolated from the x‐intercept of the linear tangent of the curve. In these OTFTs, this relationship deviated from ideality and the square root of I_D_ measured above the threshold was not completely linear, to account for this, the linear tangents of the curves were taken for the same drain current range for each OTFT (Figure S3, Supporting Information). Due to the non‐ideal electrical characteristics, the analysis focused on the trends in the parameters rather than the absolute calculated values. The mobility was calculated using the slope of the tangent line and Equation (1):

(1) $$\mu =\frac{2L}{WC}\left(\frac{d\sqrt{I_{D}}}{dV_{G}}\right)$$

where *L* and *W* are the length and width of the channel, respectively, and C is the total capacitance of the dielectrics (PS and SiO_2_), which has been previously measured for these OTFTs.^[^ ^29^ ^]^ Very minimal changes were observed in the mobilities of the OTFTs in these dose ranges, but a consistent shift in the threshold voltage was shown with dose (Figure S4, Supporting Information). This supports the theory that the main signal generation mechanism using accumulated‐dose mode is the trapping and de‐trapping of charges and not from defect creation. If defect generation was driving the shift in transfer characteristics with dose, a measurable change in mobility would be expected in addition to the shift in threshold voltage.

Figure 2c shows the real‐time response of the OTFT while being irradiated with the 3 filter conditions for 3 repetitions each. The inset shows how the current changes when the beam turns on. The real‐time response was also measured for the Al–Al filter condition, but with each dose administered being ≈235 Gy, the response of the OTFT was affected by saturation, impacting the repeatability with each repetition (Figure S5, Supporting Information). From the raw real‐time data, the integral of the current when the beam was on was calculated to determine the collected charge and divided by the administered dose to get the sensitivity (nC Gy^−1^) for each filter condition and therefore variation of the response as a function of the average energy of the photon beam. The sensitivity of the OTFT showed a slight increase with decreasing energy, shown in Figure 2d. Normalized to the lowest response ((10.3 ± 0.1) nC Gy^−1^) at 124 keV the OTFT showed a 1.6‐fold increase at 82.9 eV. Energy independence in a detector is a highly desirable characteristic as it would obviate the need for different calibration factors for different exposure (i.e., energy) conditions. One of the expected benefits of using organic semiconductors is their density and atomic composition, replicating that of the human body.^[^ ^5^, ^37^ ^]^ However, other components in the OTFT besides the organic active layers can contribute to energy dependence.

To interpret this increase in response, Monte Carlo‐based Geant4 simulations were performed using validated phase space files of the same filter conditions and experimental setup at the Australian Synchrotron.^[^ ^51^, ^52^, ^53^, ^54^ ^]^ The simulation geometry of the OTFT replicated a single‐transistor setup in the same conditions of our experimental apparatus. Interactions between the incident beam and OTFT in Solid Water HE (Gammex) were modelled to calculate the energy deposited within the pentacene layer. Simulations suggested that the dose deposited in the pentacene layer is expected to increase with decreasing energy and showed a similar trend to the experimental results, as shown in Figure 2d. However, the experimental results were higher than the corresponding simulation data for the Al‐Cu filter (82.9 keV). The discrepancy may indicate that there is also a dose rate dependence in addition to an energy dependence that is increasing the response of the OTFT with the Al‐Cu filter. Dose rates are not accounted for in the simulations, where experimentally we observe a 17‐fold increase in dose‐rate from 29.5 Gy s^−1^ with Mo‐Mo filtration to 509 Gy s^−1^ with Al‐Cu filtration. Dose rate dependencies have been reported previously with organic diodes in the same beamline^[^ ^55^ ^]^ and in clinically relevant conditions.^[^ ^14^ ^]^

To further investigate the source of the energy dependence, simulations were also carried out replacing the gold contacts in the OTFT with water and a conductive organic alternative, the poly(3,4‐ethylenedioxythiophene) polystyrene sulfonate (PEDOT:PSS). PEDOT:PSS contacts were simulated as thicker than gold at 100 nm to replicate manufacturing constraints. For comparison, simulations were also performed replacing the entire OTFT device with water to demonstrate the ideal trend with energy. As shown in Figure 2d, replacing the gold top contacts of the OTFT reduced the dose deposited in the active layer for lower energies and improved the observed energy dependence. The high Z gold increases the photoelectric effect cross section at lower energies, increasing the number of secondary electrons generated that contribute to the measured current. However, even with organic/water contacts, there was still an increase compared to the response in water, likely from backscatter of the silicon substrate. These results suggest that even the smallest quantity of high atomic number material can be detrimental for accurate dosimetry, but this effect can be reduced by replacing the gold electrodes to a lower Z organic material, such as PEDOT:PSS, and provide improved energy dependence for this range of energies. This energy dependence can be further reduced by fabricating OTFTs on a plastic substrate, such as polyethene terephthalate (PET) or Kapton.

The real‐time read‐out was also used to measure a percent depth dose (PDD) curve with the Mo‐Mo filtration. PDDs are important clinical measurements use to characterize the relative dose as a function of depth, usually in water. Measurements were normalized to the reference depth of 20 mm. Responses were measured from 5 to 60 mm depth in the solid water phantom (Figure 2e). The response of the OTFT was compared with a PTW microDiamond detector^[^ ^49^ ^]^ and a hydrogenated amorphous silicon (a‐Si:H) diode^[^ ^56^ ^]^ under the same conditions as a function of depth. The OTFT was within 8% of the microDiamond response and within 6% of the a‐Si response, confirming its functionality as a dosimeter in comparison with a clinically accepted commercial device.

The mobility was calculated for the same OTFT over the course of the synchrotron experiments at accumulated doses of 0, 100, and 600 Gy, shown in Figure 2f. These mobilities were calculated from a pristine OTFT at the beginning of subsequent days. The OTFT showed a decrease in mobility with dose, which is evidence of radiation damage and defect generation in these materials. To confirm this trend with higher doses, mobility was calculated from an OTFT that was irradiated with a linear accelerator (6 MV FFF) to 2000 Gy and left to rest a week. A decrease in mobility of 20% was calculated for this OTFT (Figure 2f), but the threshold voltage returned to the same voltage as before irradiation (Figure S6, Supporting Information). For reference, in radiation therapy conventional dose delivered in a fraction is ≈2 Gy, a high fraction of dose would be 10–20 Gy (with FLASH, for example). After 100 Gy, the OTFT showed a decrease in mobility of 2.25%, suggesting that for clinically relevant dose ranges, the mobility would be stable. To further investigate the exhibited decrease in mobility and the associated underlying mechanisms of radiation damage in these OTFTs, the materials were characterized using soft x‐ray spectroscopy techniques to investigate the individual materials and the combination of pentacene and polystyrene.

### 2.2 XPS and NEXAFS Analysis of Pristine and Irradiated Materials

**Figure 3:**
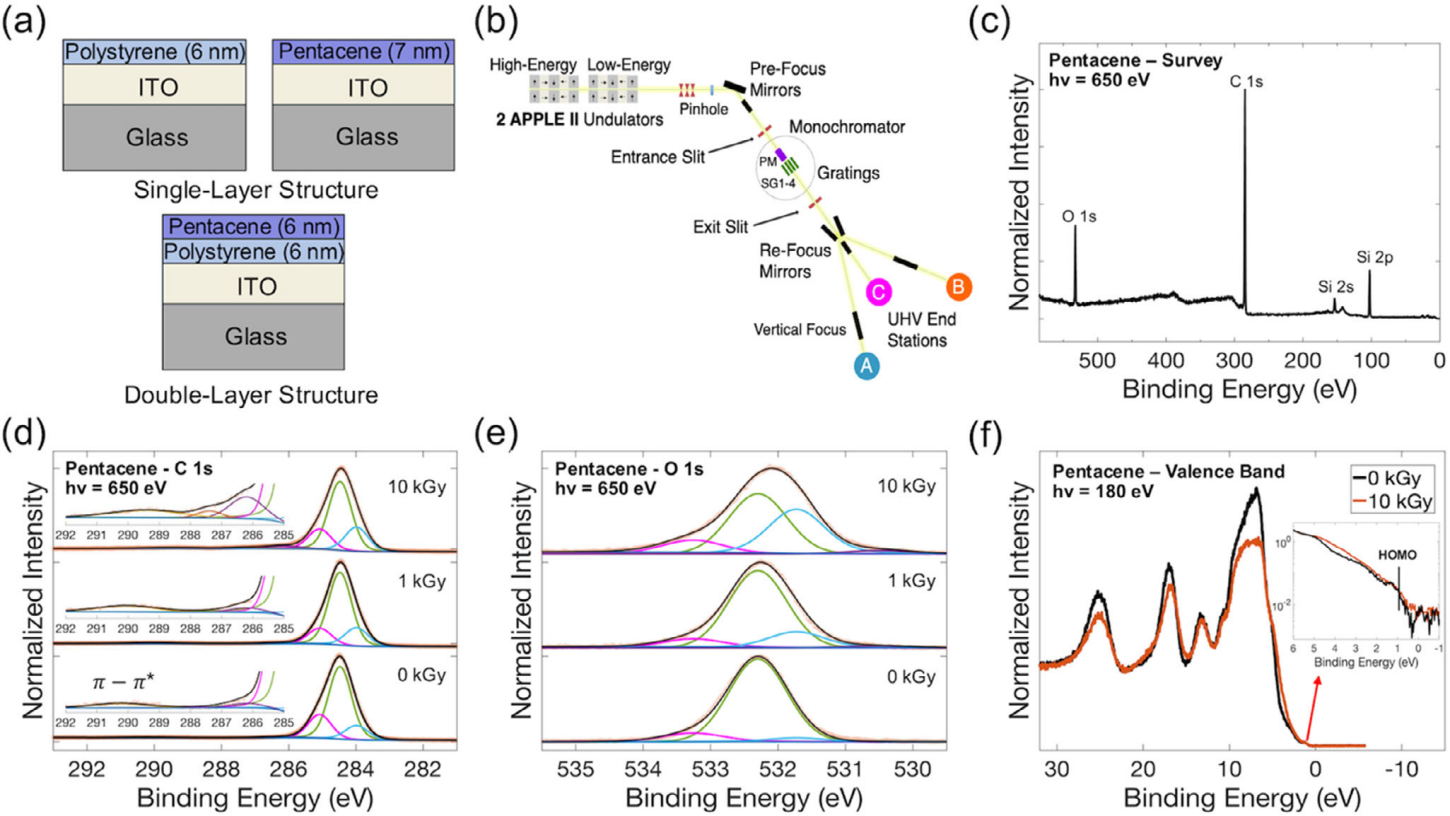
a) Schematic of samples used for XPS and NEXAFS analysis with individual materials used in single‐layer structure and both materials in a double‐layer structure. b) Schematic of BACH beamline at Elettra. c) XPS survey spectrum for pristine pentacene sample, normalized to maximum. XPS High resolution spectra of: d) C 1s core levels (normalized to maximum) for pristine, 1 and 10 kGy doses, insets show magnification of the *π*–*π* ^*^ shake‐up satellites and the carbon‐oxygen bond energy region; e) O 1s core levels (normalized to maximum) for pristine, 1 and 10 kGy doses; f) valence band (normalized to area) for pristine, and 10 kGy dose.

To gain insight into how radiation affects the materials in these OTFTs, XPS and NEXAFS techniques were used to investigate the chemical and electronic properties of pristine and irradiated materials. Pentacene and PS films were fabricated in single‐ and double‐layer structures on Indium‐Tin‐Oxide (ITO) glass, as shown in **Figure** **3**a. Materials and fabrication techniques were the same as those used in the OTFTs, and the stack of thin films closely resembled the architecture of the active layers in the OTFT. For each material, one sample was left pristine, one was irradiated to a total dose of 1 kGy, and another to a total dose of 10 kGy. Samples embedded in a plastic 1 cm thick enclosure, were irradiated with cobalt‐60 gamma rays exposing the samples to a mixed radiation field. In this energy range, the primary photon interaction with low‐Z materials is Compton scattering, which generates secondary electrons. A small percentage of electrons will also be generated via the photoelectric effect. These secondary electrons lose energy to the surrounding medium as they travel through the material, primarily through inelastic collisions with other electrons, ultimately coming to rest and depositing their remaining energy locally.^[^ ^57^, ^58^ ^]^ These radiation conditions have been chosen to introduce radiation damage from a mixed field and because Co‐60 presents a gamma photopeak approximately at the same energy of the average energy of a 6 MV medical linear accelerator.

Both XPS and NEXAFS techniques were performed at the BACH beamline at the ELETTRA synchrotron,^[^ ^59^, ^60^ ^]^ as shown is Figure 3b. Samples were measured in ultra‐high vacuum (UHV) conditions. Single‐layer structures on ITO‐glass were first characterized with XPS (hν = 650 eV). The binding energy (BE) scale calibrations were carried out by measuring Au 4f_7/2_ for gold foil in electric contact with the sample on each sample holder, and corrected the peak position to 83.95 eV (Au 4f_7/2_ BE).^[^ ^61^ ^]^ From the survey spectrum, carbon, oxygen, and silicon elements were detected in all samples, as demonstrated by the survey scan for pristine pentacene shown in Figure 3c. Pentacene and PS only contain carbon and hydrogen and do not contain silicon or oxygen, though a small amount of oxidation is expected from a limited exposure to the atmosphere. Most of the measured oxygen is likely due to silicon oxide (Figure S7, Supporting Information). The presence of silicon oxide on the surface can be attributed to the sample preparation process of the glass substrates and cutting them to size; dust from cutting the ITO‐glass was not completely removed from the surface, or the ITO was scratched, resulting in exposed glass material on the sample's surface (Figure S8, Supporting Information). Although the presence of silicon complicated the analysis, its contributions were subtracted to clearly identify trends in the data with increasing dose.

To attain more detailed information about the chemical structure, high‐resolution photoemission spectra were collected for individual features. Specifically, C 1s, O 1s core levels, and valence band were the data of interest. In fitting all core‐level peaks, pristine sample data were fit first, and the relative BEs, Gaussian/Lorentzian contributions, and peak widths were used for the irradiated data, adding slight variations and additional peaks when necessary. Figure 3d shows the C 1s of pentacene for pristine and irradiated samples. The main peak position (284.4 eV) is in good agreement with previous reports of pentacene thin films.^[^ ^62^, ^63^ ^]^ In the literature, XPS measurements and modelling of pentacene have shown that C 1s peak is asymmetric, with a main peak assigned to “un‐hydrogenated” carbons and a lower BE shoulder assigned to the rest of the carbons in the pentacene molecule.^[^ ^63^, ^64^ ^]^ The C 1s peaks of our thin film pentacene samples are more symmetric, and their best fits were obtained using 3 main components, similarly to the fits reported by Nakayama et al. for pentacene thin‐films on Au.^[^ ^63^ ^]^ Another small peak was added to fit to higher BE tail of the C 1s peak, which is ascribed to C—O functional groups (+1.2–1.5 eV with respect to the main carbon peak).^[^ ^65^, ^66^ ^]^ This tail was affected by irradiation and increased with dose, enhancing the contribution of C─O bonds. Another effect of irradiation is the presence of an additional peak which, according to literature, can be associated with C═O (+3–4 eV).^[^ ^65^, ^66^ ^]^

The increase in C─O bonds was also confirmed in the O 1s peak of pentacene, shown in Figure 3e. In the pristine pentacene O 1s, the main peak can be attributed to silicon oxide with a BE of 532.3 eV, in agreement with the literature.^[^ ^65^, ^67^ ^]^ Further confirmation that the silicon presence was due to traces of silicon oxide is from the Si 2p BE of 102.3 eV, which is higher than that of pure silicon (99.5 eV).^[^ ^68^ ^]^ According to previous studies, the BE for SiO_2_ is 103.5 eV with intermediate oxidized silicon states found at BEs between Si and SiO_2_,^[^ ^69^, ^70^ ^]^ suggesting the silicon present was of a silicon‐oxygen environment. The other components of the pristine pentacene O 1s peak can likely be attributed to some oxidation of the pentacene sample from a limited exposure to air, C─O functional groups for aromatic carbon have a BE of ≈533 eV,^[^ ^65^, ^67^ ^]^ which is in good agreement with the higher BE shoulder peak (533.2 eV). The lower BE component (531.7 eV) can be ascribed to C═O functional groups.^[^ ^65^, ^67^ ^]^ As with all core‐level peaks shown, the fitting parameters were established from the pristine sample. With increasing dose, the O 1s peak becomes broader and slightly shifted toward lower BE, indicating that oxygen atoms occupy multiple chemical environments after irradiation. The peaks attributed to C─O and C═O functional groups both increase with dose, C═O more than C─O. The relative areas for C─O and C═O at 0 kGy were 0.10 and 0.04, respectively and increased to 0.12 and 0.38 after 10 kGy. This corresponds to a C═O/C─O ratio increase from 0.40 to 3.17. An additional peak at the lower BE tail was also added, ascribed to the oxygen of the ITO substrate.^[^ ^71^ ^]^

The valence band was measured using lower energy incident photons (180 eV) corresponding to an electron inelastic mean free path of ≈0.7–0.8 nm (or a sampling depth of ≈2–2.5 nm) to avoid contribution from the substrate. Figure 3f demonstrates the valence band of pristine and 10 kGy pentacene. The lower BE region was not well resolved as it is dominated by the O 2p signal from the silicon oxide from ≈5–10 eV.^[^ ^72^ ^]^ However, from this region, we again observed a change in the oxygen environment from pristine to irradiated samples. The inset of Figure 3f shows magnification of the lowest BE range, where a slight broadening of the HOMO and other filled orbitals occurs after irradiation. The absence of any additional peaks above the HOMO edge suggests there is no significant creation of states in the bandgap from radiation. If any states were created, they are at a density lower than the detection limit of XPS, on the order of 1%. The pentacene single‐layer structure results indicate that radiation exposure leads to some C─H and C─C bonds in the molecule breaking and becoming oxidized. Oxidation of pentacene has been shown to degrade field effect mobility in OTFTs,^[^ ^73^ ^]^ which suggests that the reduction in mobility observed in our OTFTs following high radiation doses (Figure 3f) may be due to increased oxidation of the pentacene.

**Figure 4:**
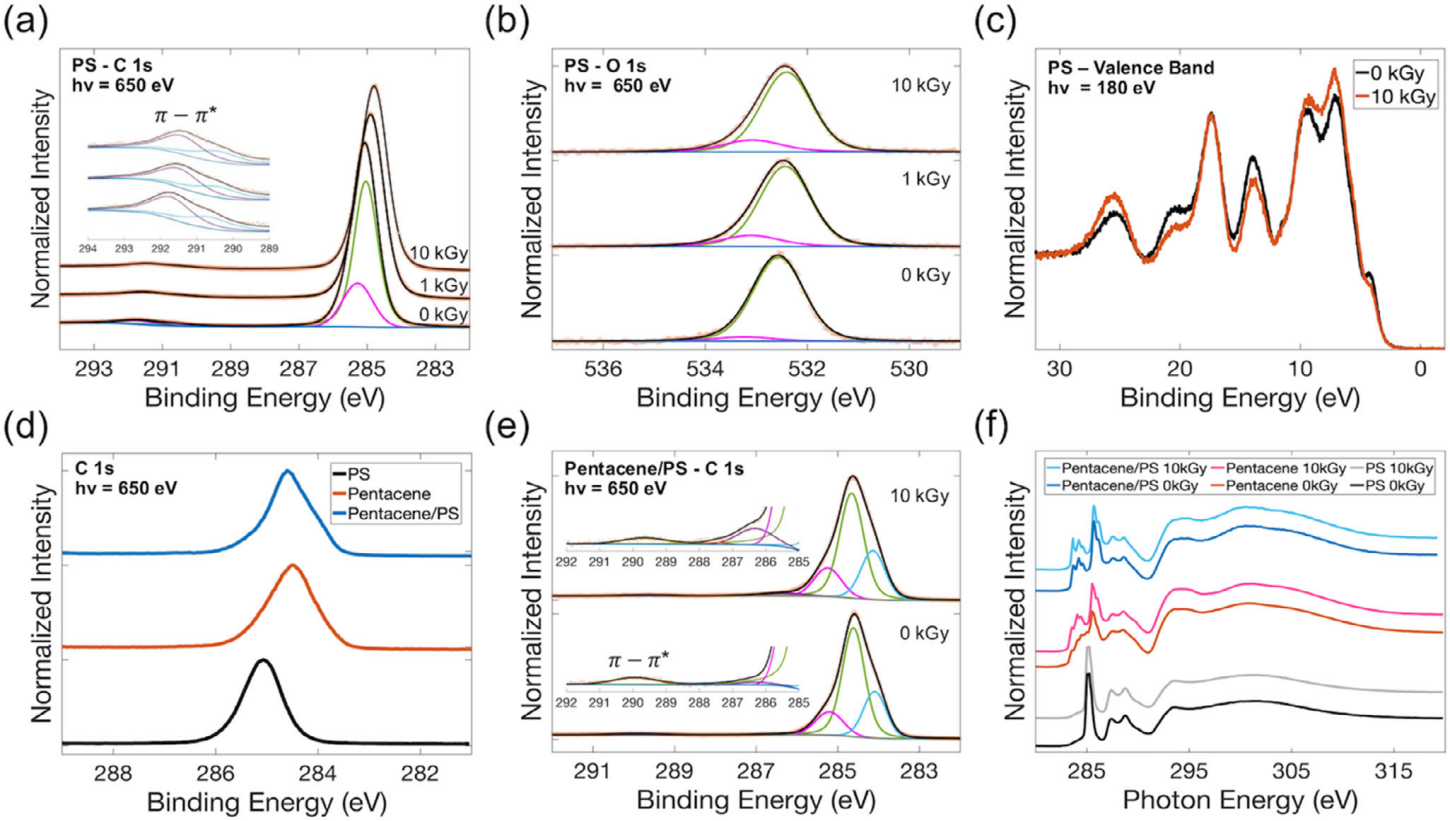
High resolution XPS spectra of pristine and irradiated single‐layer polystyrene: a) C 1s core level (normalized to maximum), inset shows magnification of the *π*–*π* ^*^ shake‐up satellites; b) O 1s core level (normalized to maximum) and c) valence band (normalized to area). d) C 1s spectrum of pristine double‐layer sample (Pentacene/PS) in comparison with single‐layer samples of the individual materials (normalized to maximum). e) C 1s spectrum of double‐layer sample for pristine and 10 kGy irradiated samples (normalized to maximum), insets show magnification of the *π*–*π* ^*^ shake‐up satellites and the carbon‐oxygen bond region. f) Carbon K‐edge NEXAFS spectra of all pristine and irradiated samples (normalized to maximum).

The C 1s spectra for polystyrene single‐layer samples is shown in **Figure** **4**a. The C 1s envelope of polystyrene was fit by 2 components ≈0.24 eV apart (Figure S9, Supporting Information), the main peak corresponds to the aromatic carbon and the higher energy peak corresponds to the backbone carbon in the material.^[^ ^65^, ^74^ ^]^ The C 1s peak shifted to lower BEs with dose, the aromatic carbon peak shifted from 285.04 to 284.74 eV, a change of 0.3 eV from pristine samples to 10 kGy irradiated films. This shift could indicate a shift in the Fermi level of the material. The BEs reported are in reference to the Fermi level of the material; if the Fermi level shifts, so will the BEs of the elemental peaks. The shift to lower BEs of the C 1s corresponds to the Fermi level moving toward the highest occupied molecular orbital (HOMO) and is consistent with the creation of acceptor‐like defect states or p‐doping of the material.^[^ ^75^ ^]^ Creation of defect states in the PS would also explain the observed decrease in OTFT mobility after irradiation. These acceptor defect states can trap electrons and increase scattering of mobile charge carriers in the OTFT, reducing free carriers in the channel.^[^ ^76^ ^]^ This shift indicates that the dielectric properties of the material may also be changing. Raval et al. reported a similar shift in the C 1s BE of pentacene with a 100 Gy dose of gamma irradiation and showed a decrease in the resistance of a pentacene‐based resistor by a factor of 5 after 100 Gy.^[^ ^77^ ^]^ Meaning the material is less insulating and would increase current leakage, degrading the charge retention capabilities in the electret OTFT. However, it has been shown in polypropylene (PP) capacitors that their capacitance remains unchanged after 100kGy of gamma irradiation.^[^ ^78^ ^]^ PP is generally considered to be less radiation resistant than polymers containing benzene rings, such as PS, therefore, we would expect minimal changes in the capacitance of PS after 10 kGy. Furthermore, in the OTFT the total dielectric capacitance is largely determined by the thicker SiO_2_ layer, therefore variation in the PS capacitance would only have a minor impact on the overall capacitance of the OTFT.

Pristine PS O 1s were fit similarly to the pentacene O 1s, most of the peak was made up of the silicon oxide, with another component from the oxidation of the material, as shown in Figure 4b. The C─O peak showed a slight increase with dose, but remained relatively unchanged compared to the pentacene's oxygen environment, and the C═O contribution is not observed. The valence band spectra for pristine and 10 kGy PS are shown in Figure 4c. They show a good agreement with the literature results of spin‐coated PS film on Si,^[^ ^79^ ^]^ except for the peak at ≈26 eV, which was also present in the pentacene valence band. This peak is attributed to O 2s.^[^ ^48^, ^49^ ^]^ Small variations were observed in the valence band structure, most interestingly, the lowest BE peak at ≈5eV, which can indicate a slight widening in the bandgap.

Figure 4d shows the C 1s spectra of the pristine stacked pentacene/PS in comparison with the individual materials. The signal in the double‐layer structure is expected to mostly be from the pentacene as it is deposited above, with some contribution from the PS underneath. Comparison of the C 1s peaks showed that the signal from the pentacene/PS stack most closely resembled that of pentacene, but it was more asymmetric and had a slightly shifted BE. This can either be from the contribution of the PS to the signal and/or the pentacene was organized differently on the PS vs ITO. Pentacene is known to either “stand up” or “lay flat” on the surface depending on the underlying substrate.^[^ ^80^ ^]^ The C 1s peak for the pentacene/PS sample was more asymmetric than the pentacene sample and is in better agreement with other C 1s spectra reported for pentacene films reported in literature.^[^ ^63^ ^]^ Similar to the single‐layer pentacene sample, the C 1s peak was fit with 3 components, with more contributions from the lower BE shoulder peak, as shown in Figure 4e. The increase in the higher BE tail from C─O and C═O functional groups, indicating some oxidation of the material with radiation, was also confirmed in the stacked sample.

The carbon K‐edge NEXAFS spectra were also measured for all samples at the “magic angle”, at which the polarization vector of the incident X‐ray beam is at ≈55° with respect to the surface normal. At this incident angle, the NEXAFS resonant intensities are independent of the molecular orientation on the surface.^[^ ^81^ ^]^ Figure 4f shows the NEXAFS spectra for pristine and 10 kGy samples for each material. The PS spectrum agrees well with previous measurements in the literature.^[^ ^42^, ^79^ ^]^ The peak assignments of polystyrene are similar to that of benzene^[^ ^82^ ^]^: the first peak at 285.1 eV and third peak at 288.7 eV are assigned to transitions of core electrons into unoccupied π^*^ states, the second peak at 287.4 eV is assigned to 3p C—H^*^/Rydberg state, and the broader peaks at 293.5 and 302 eV are σ^*^ transitions. No changes induced by radiation are observed in the PS spectrum. Pentacene NEXAFS resonances between 283 and 287 eV correspond to π^*^ transitions of the sp^2^‐hybridized carbon atoms, between 287 and 291 eV C─H^*^/Rydberg states, and between 291 and 315 eV σ^*^ transitions.^[^ ^42^, ^83^, ^84^ ^]^ A Summary of peak assignments for pentacene and PS are shown in **Table** **2**. C K‐edge spectrum for pentacene/PS sample varied from the one measured for the pentacene single‐layer sample, indicating either signal contributions from both materials and/or pentacene molecules arranged differently when grown on PS. Variations were observed in the relative intensity of the π^*^ peaks in pentacene and pentacene/PS after irradiation. However, different angles of incidence were also investigated for the pentacene single‐layer sample and similar variations in the π^*^ region were observed (Figure S10, Supporting Information). Thus, it cannot be concluded that these changes are due to radiation damage, as they can be from a different ordering of the pentacene molecule on the substrate from sample to sample. NEXAFS spectrum reveal no major changes in the carbon chemical bonds/electronic structure, specifically they do not reveal additional states or defects generated with radiation damage in these materials. Anbalagan et al. reported a small increase in the π^*^ unoccupied states in pentacene after irradiation with NEXAFS, but changes in the overall spectrum were also minimal.^[^ ^84^ ^]^

**Table 2:** Summary of NEXAFS peak assignments for pentacene and PS thin film samples.

Material	Peak energy [eV]	Transition type
Polystyrene	285.1	1s → π^*^
	287.4	1s → 3p Rydberg
	288.7	1s → π^*^
	293–302	1s → σ^*^
Pentacene	283–287	1s → π^*^
	287–291	1s → C—H^*^/Rydberg
	291–315	1s → σ^*^

## 3 Conclusion

Polymer electret OTFTs have demonstrated potential in radiation dosimetry applications for ultra‐high dose rate environments. OTFTs were tested in two readout modes, accumulated‐dose and real‐time, for three filter conditions of the synchrotron beam. They exhibited excellent linearity with all filters with a maximum sensitivity of (21.0 ± 0.5) mV Gy^−1^ in accumulated‐dose readout. With real‐time response, an increase in sensitivity with decreasing energy was observed. Geant4 simulations revealed that the gold electrodes are the main source of the response variation with energy, and replacing contacts with a lower Z material can improve energy dependence. Discrepancies between experimental and simulation results at Al‐Cu filtration also suggest that there is a dose‐rate dependence as well. It was also demonstrated that these OTFTs are capable of PDD measurements in good agreement with commercially available detectors.

Minimal variation in the mobility of the OTFTs paired with a consistent shift in threshold voltage with low doses supports a trapping model for the main signal generation mechanism. After larger irradiation doses, a reduction in the mobility of the OTFT becomes apparent. XPS analysis revealed that when irradiated with high doses, some carbon bonds in pentacene are breaking, leading to an increase in oxidation with dose, which can explain the reduction in mobility in the OTFTs. It was demonstrated that in PS, radiation damage can manifest in a downshift in the Fermi level, which is consistent with creating acceptor‐like defects that would also reduce mobility in the OTFTs. The pentacene/PS stack showed less oxidation with dose compared to pentacene on ITO. The PS likely creates a better environment for the pentacene to be deposited, which was also suggested by the NEXAFS spectrum. NEXAFS spectrum revealed no significant variation in the electronic structure of these organic materials with radiation damage.

## 4 Experimental Section

### OTFT Fabrication

OTFTs were fabricated in a top contact/bottom gate configuration on heavily doped Si/SiO_2_ substrates. PS (Sigma‐Aldrich, Mw = 280 kg mol^−1^) was used to make a solution in toluene and spin‐coated on top of SiO_2_ to form a 45 nm additional dielectric layer. Pentacene (TCI America) was thermally evaporated through a shadow mask to a thickness of 50 nm. Gold source and drain interdigitated electrodes were also thermally evaporated on top of the pentacene. OTFTs were encapsulated in parylene‐C. The device fabrication process has been described in more detail in previous work.^[^ ^29^ ^]^

### Electrical Characterization

OTFTs were characterized with a dual‐channel SMU (Keithley 2614B, Tektronix, Inc.). OTFTs were used to measure dose in two readout modes, accumulated‐dose and real‐time. Connections for the two readout methods are shown in Figure 1c, in accumulated‐dose mode a high bias of −80 V was applied to the gate for 3 s prior to irradiation, this temporarily shifted the threshold voltage negatively and shifted the *I*–*V* characteristics.^[^ ^29^ ^]^ A transfer curve was measured holding V_D_ at −20 V and sweeping V_G_ from 0 to −20 V, the OTFT was then irradiated with no bias applied. Immediately after irradiation, another transfer curve was taken to quantify the shift from pre‐ to post‐irradiation. In real‐time readout, −20 V was applied to the gate and drain, and the drain current was measured as a function of time as the OTFT is irradiated. OTFTs were placed in a 3D‐printed polylactide (PLA) sample holder during irradiations/readout, which has been shown to be water equivalent at the IMBL.^[^ ^85^ ^]^ Metal spring‐pins were used for electrical contacts, and triaxial cables were used to connect the OTFTs to the SMU, which was housed outside of the hutch.

### Synchrotron X‐ray Characterization

X‐ray measurements were conducted with the IMBL at the Australian Synchrotron.^[^ ^46^, ^47^, ^48^ ^]^ Synchrotron radiation was produced by a 3T wiggler magnetic field, and experiments were performed in hutch 2B located 32 m away from the wiggler source. The average energy, dose rate, and flux of the beam were altered with a combination of filters (Mo, Cu, Al). The beam was shaped with a beam defining aperture of 1.052 cm height and 30 mm width, then with a conformal mask of 20 × 20 mm^2^. The OTFT was placed in a 10 × 10 × 10 cm solid water phantom at a reference depth of 20 mm. The OTFT, phantom, and mask were translated in the z‐direction through the 20 × 20 mm^2^ field via a motorized stage known as the “DynMRT” stage. A PTW PinPoint Ionization chamber was used for reference dosimetry.

### OTFT Detector Characterization

In the accumulated‐dose readout mode, linearity was assessed for Mo‐Mo, Cu‐Cu, and Al‐Cu filter conditions. For Mo‐Mo scans were conducted at a speed of 10 mm s^−1^ and for Cu‐Cu/Al‐Cu scans were at a speed of 20 mm s^−1^. In real‐time readout mode, measurements were taken for all filter conditions at 20 mm s^−1^, and PDD measurements were taken for the Mo‐Mo filter at 10 mm s^−1^.

### Geant4 Simulations

Monte Carlo simulations were performed using Geant4 Version 11.00‐patch‐03 with the Livermore low‐energy electromagnetic physics model. The Geant4 simulations used phase space files generated from the detailed and validated model of the IMBL at the Australian Synchrotron from the wiggler onward.^[^ ^51^, ^54^, ^86^ ^]^ Phase space files were generated using the same filter conditions as the experiment to an area of the same field size, 20 × 20 mm^2^. The geometry of the OTFT and experimental set up was replicated. The energy deposited per primary particle in the 50 nm pentacene layer was calculated for each filter condition.

### XPS/NEXAFS Samples Fabrication

Pentacene and polystyrene films were fabricated in single‐ and double‐layer structures on ITO‐glass. Materials were the same as used in the OTFTs, and the thin film structures closely resemble the architecture of the active layers in the OTFTs. ITO‐glass slides were first cut into 10 × 10 mm^2^ pieces and consecutively sonicated in acetone, isopropyl alcohol, and deionized water for 10 min each. Samples were then placed under UV‐ozone for 15 min. 2 mg ml^−1^ solution of polystyrene in toluene was spin‐coated onto the ITO substrate at 6000 rpm for 60 s, samples were dried at 90 °C for 1 h. For pentacene single‐layer samples, the pentacene was thermally evaporated at 10^−6^ torr at a rate of ≈1 Å/s onto the ITO. For the double‐layer structures, the PS was spun onto the ITO first with the same procedure, then the pentacene was thermally evaporated on top of the PS layer. The thickness of the films on the substrates was estimated with atomic force microscopy, by wiping away the polystyrene from an edge after spinning and covering a portion of the substrate during pentacene evaporation to create a step. For each material, one was left pristine, one was irradiated to a total dose of 1 kGy, and another to 10 kGy with a cobalt‐60 gamma source. Samples were made prior to the XPS/NEXAFS measurements and were stored and transported in vacuum sealer bags that were heat sealed under a nitrogenous atmosphere to prevent degradation from the atmosphere as much as possible.

### XPS and NEXAFS Measurements

Measurements were done at the CNR beamline BACH at the Elettra synchrotron radiation facility in Trieste, Italy.^[^ ^59^, ^60^ ^]^ The measurements were performed in UHV at a base pressure of 10^−9^ mbar. Data was acquired using a VG‐Scienta R3000 hemispherical analyzer. XPS data were collected with incident photon energies of 180 and 650 eV, with 0.15 eV and 0.2 eV total energy resolution, respectively. Carbon K‐edge NEXAFS spectra were collected in total electron yield mode. Intensities were normalized to the incident photon flux, measured by the drain current on a reference golden grid. The energy resolution was set to 0.1 eV in the energy range used.

### Statistical Analysis

For the accumulated‐dose response, *I*–*V* curves and voltage shifts were measured for a single OTFT. Error bars associated with these measurements correspond to the instrument error of the Keithley 2614B SMU (0.02% + 5 mV for voltage, 0.025% + 500 pA for current) specified for 1 power line cycle (PLC). Measurements were taken with 3 PLC, therefore reported errors represent a conservative estimate. Linear regression was used to calculate slope, standard errors, and R^2^ values for accumulated‐dose response fits, The sensitivity of this OTFT response is reported as slope ± standard error. For calculated mobilities, error bars represent the propagated standard error from the slope of the square root of I_D_ above threshold. For real‐time response measurements, each irradiation condition was repeated three times on the same OTFT device. Sensitivities are reported as mean values ± standard deviation. Data analysis was done with MATLAB software. XPS peak fitting was done with the KolXPD software package.^[^ ^87^ ^]^ The C 1s and O1s spectra were fitted using Voigt functions after subtracting a Shirley background. Peak fitting was based on pristine samples, and minor adjustments/additional peaks were then added for irradiated materials.

## Conflict of Interest

The authors declare no conflict of interest.

## Supporting information



Supporting Information

## Data Availability

The data that support the findings of this study are available from the corresponding author upon reasonable request.
