# Supplementary material for: Radiation Effects in Electret Organic Thin‐Film Transistors Due to High Flux and High Dose X‐Ray Irradiation
Source: Adv Mater. 2025 Nov 29;38(8):e08402. doi: 10.1002/adma.202508402 (PMC12878810; doi:10.1002/adma.202508402)
Supplement: Supplementary file 1 — Supporting Information [file ADMA-38-e08402-s001.docx]

Supporting Information

Radiation Effects in Electret Organic Thin-Film Transistors Due to High Flux and High Dose X-Ray Irradiation

Alexandria Mitchell, Jessie A. Posar, James Cayley, Georgia York, Michael Lerch, Attila J. Mozer, Igor Píš, Elena Magnano, Luca Tosti, Maddalena Pedio, Alasdair Syme, Ian G. Hill, and Marco Petasecca*

To confirm the importance of the programming bias in the radiation response of these electret OTFTs, the OTFTs were irradiated without applying the programming bias prior to irradiation. The OTFT was placed at a depth of 5 cm in solid water and irradiated with a 6 MV linear accelerator. To read out the OTFTs, negative voltages were applied to the gate which can have a programming effect on the device by trapping charges. As shown in Fig. S1a, repeated scanning of the device without radiation caused a slight negative voltage shift. When the OTFT was irradiated without the programming bias, the same small negative shift continued with each scan. Therefore, the radiation had no effect on the response of the OTFT. When the OTFT was then programmed and irradiated, a larger positive shift in voltage was observed, confirming that the programming bias enables the OTFT’s response to radiation.

To verify that the programming effect and the associated response to radiation are due to the presence of the polystyrene electret, identical OTFT devices were fabricated without the polystyrene layer. As shown in Fig. S1b, the behavior of these devices was vastly different from that of the polymer electret OTFTs, exhibiting a large positive threshold voltage due to the well-known effect of electron trapping on the SiO₂ surface.^[1]^ The threshold voltage of these devices was unstable and repeated scans resulted in large positive voltage shifts. When irradiated, the threshold voltage continued to shift positively but showed no additional change beyond that observed during repeated scanning, indicating no detectable radiation response. Attempts were made to apply a programming bias to the devices without polystyrene but always ended up in a breakdown of the dielectric, presumably because of the large electric field already present from the electron trapping.


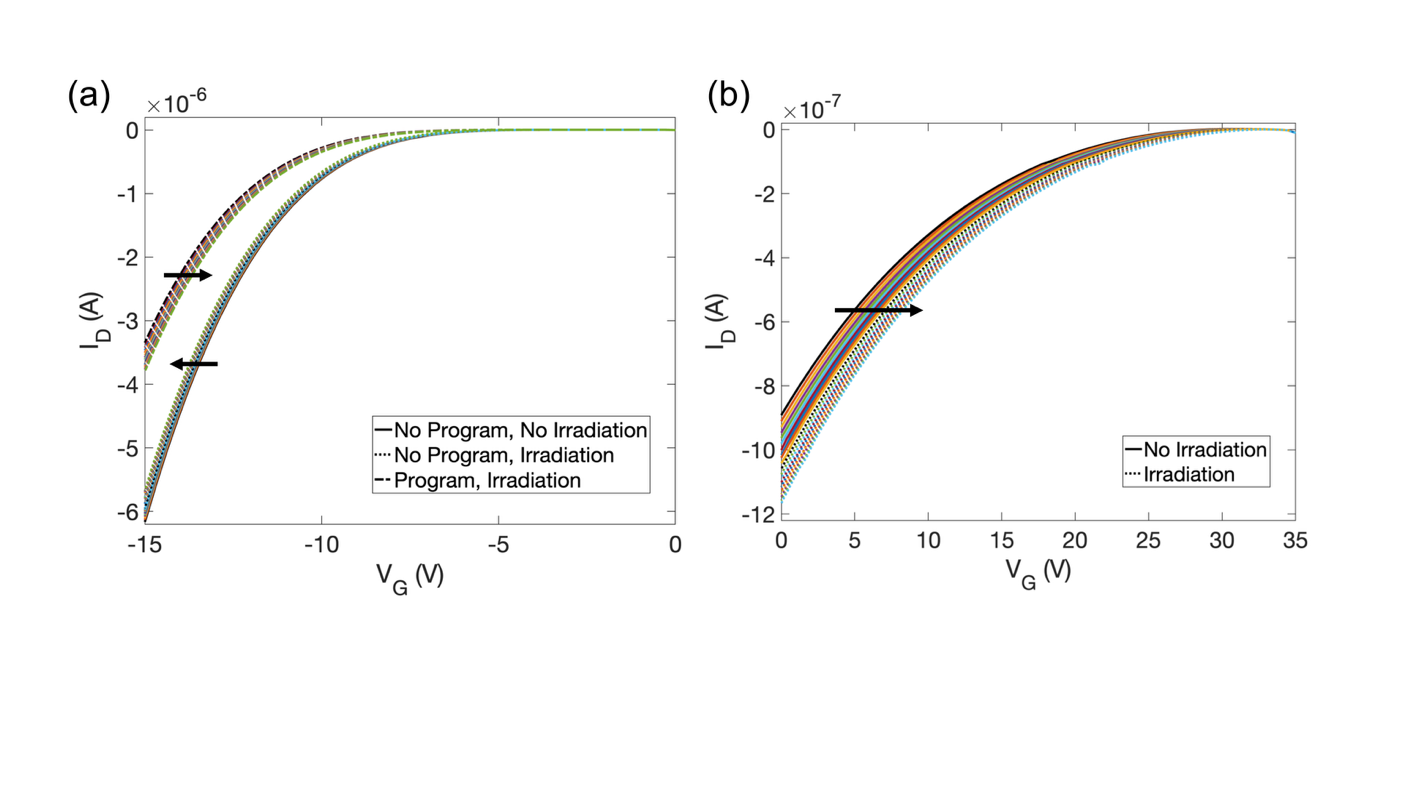


**Figure S1.** a) OTFT first scanned 10 times without programming and without irradiating, waiting 1 minute between scans to replicate time for irradiation, as shown by solid lines. Next, the OTFT was irradiated 10 times with 2 Gy dose and read out after each dose step without programming the OTFT before irradiations, as shown by dotted lines. The OTFT was then programmed with -80 V for 3 s and irradiated 10 times with 2 Gy and read out after each dose step, shown by dashed lines. b) OTFT without polystyrene layer first scanned 10 times without programming and without irradiating, waiting 1 minute between scans, as shown by solid lines. Then the OTFT without polystyrene was irradiated 10 times with 2 Gy dose and read out after each dose step without programming. Arrows represent the direction of the voltage shift after subsequent read outs.


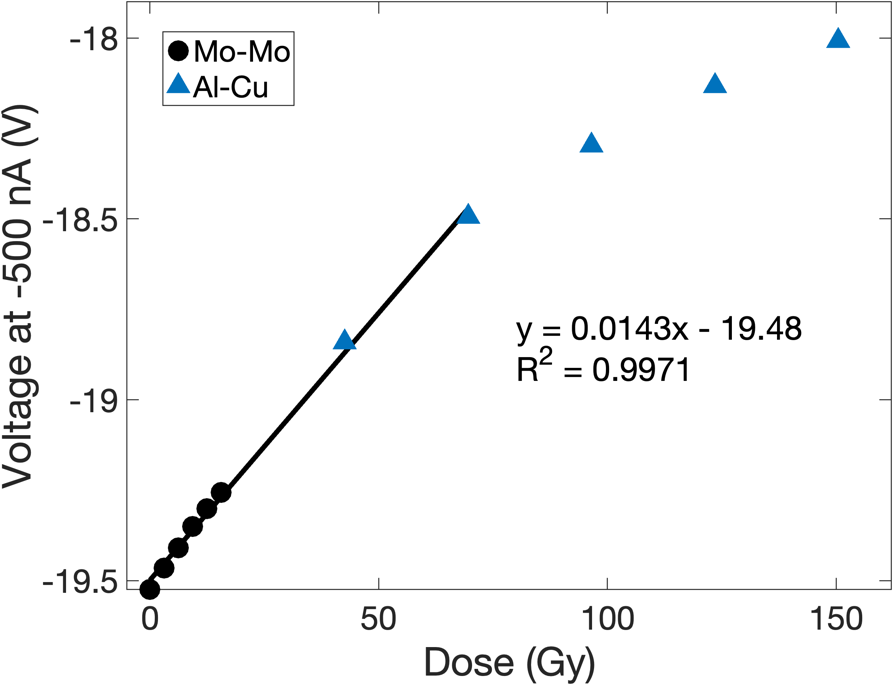


**Figure S2.** Gate voltage at -500 nA taken from OTFT transfer characteristics as a function of dose for irradiations with Mo-Mo and Al-Cu filtrations. Error bars are smaller than markers, error is from measurement error from SMU. Device response starts to saturate after 70 Gy.


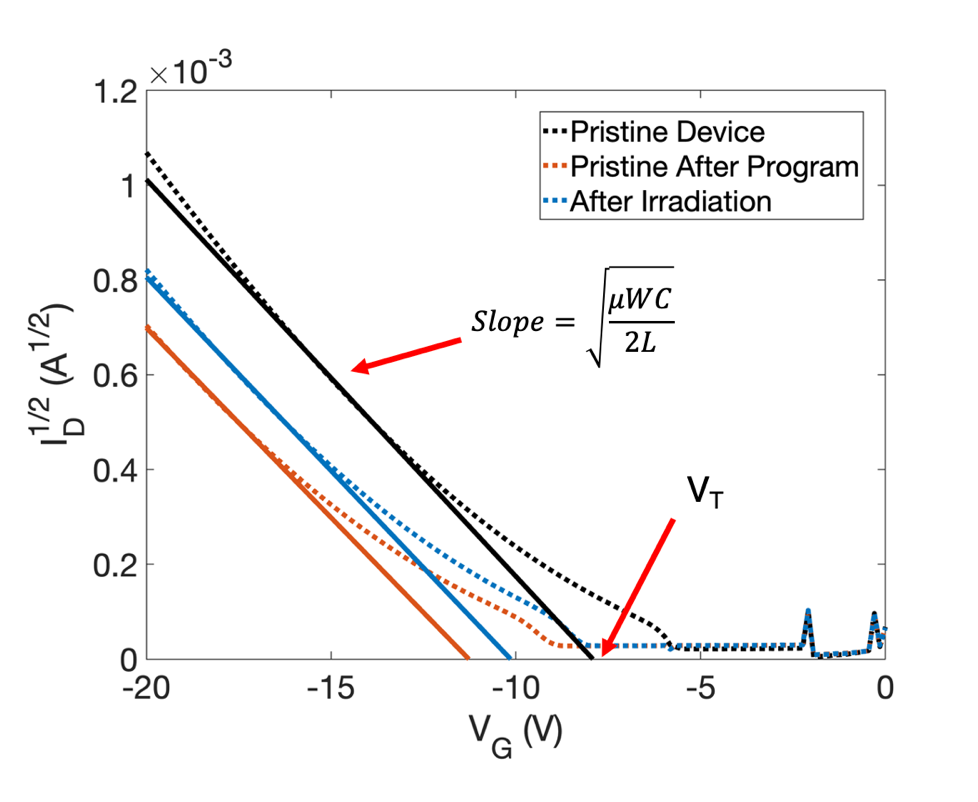


**Figure S3.** The square root of drain current as a function of gate voltage for the same device before irradiation (pristine), then programmed with -80 V for 3 s, and irradiated. Tangent lines were fitted for ± 10 data points from I_D_ = -320 nA for each curve for consistency.


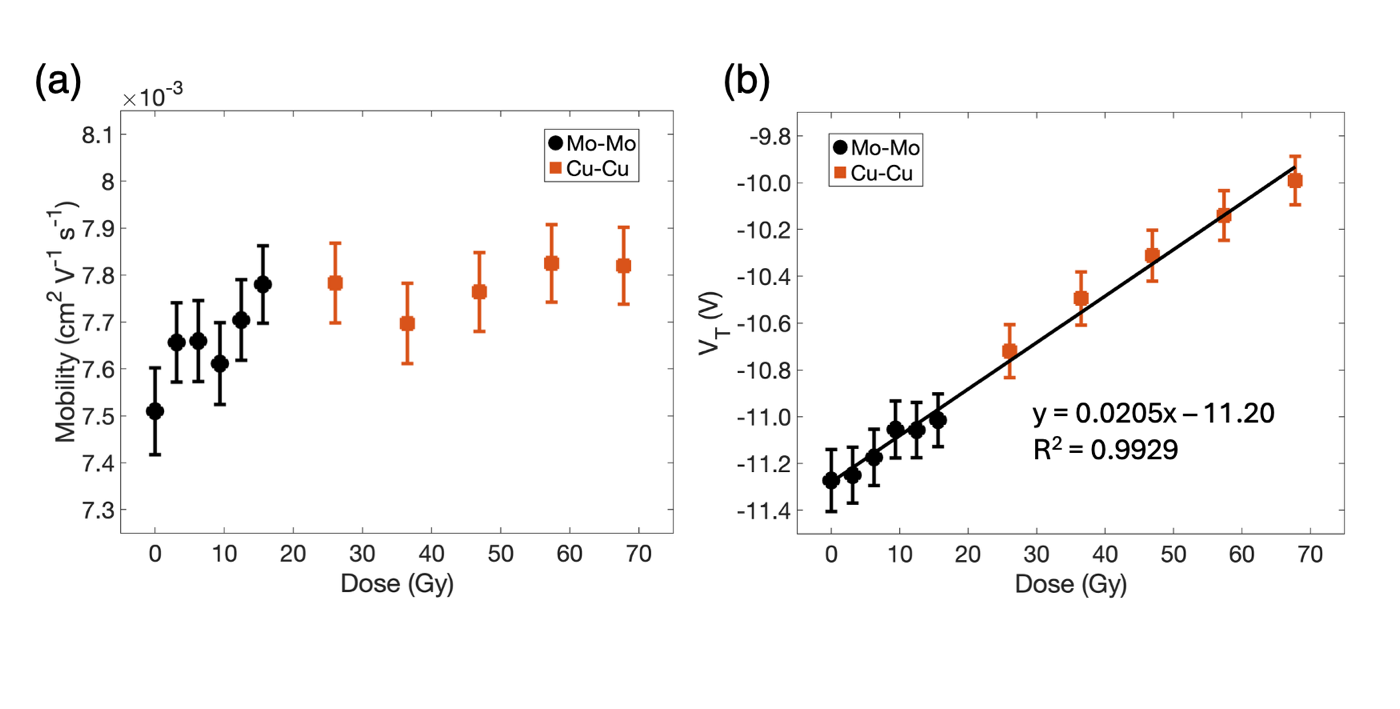


**Figure S4.** a) Mobility calculated from the transfer curves collected at each dose step for subsequent Mo-Mo and Cu-Cu irradiations as a function of dose. b) Threshold voltage calculated for the same irradiations as a function of dose.


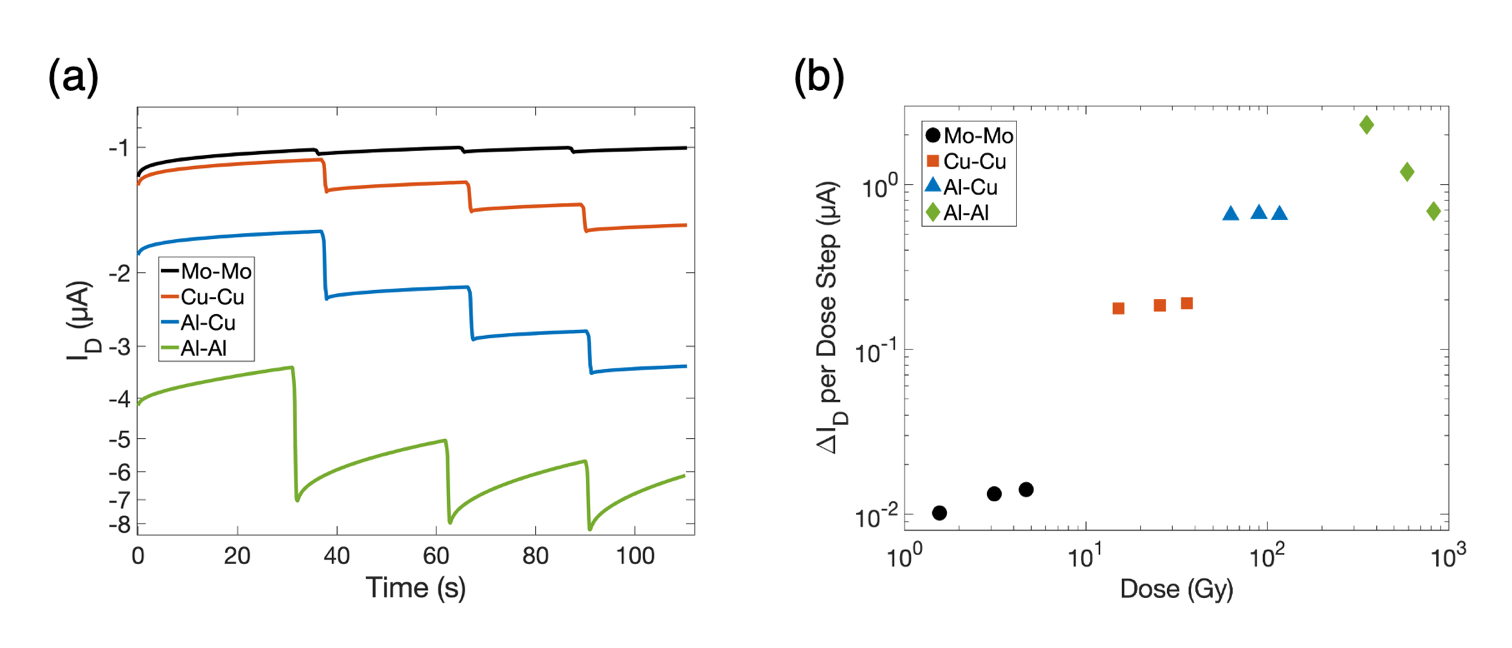


**Figure S5.** a) Real-time readout of OTFT, V_DS_ = V_GS_ = -20 V and drain current is monitored with time. Irradiation from all filtrations including Al-Al for 3 repetitions each for statistics. b) Change in drain current for each dose step for all filters, change in current for each Al-Al irradiation (235 Gy each) decreasing with dose, indicating device damage from radiation.


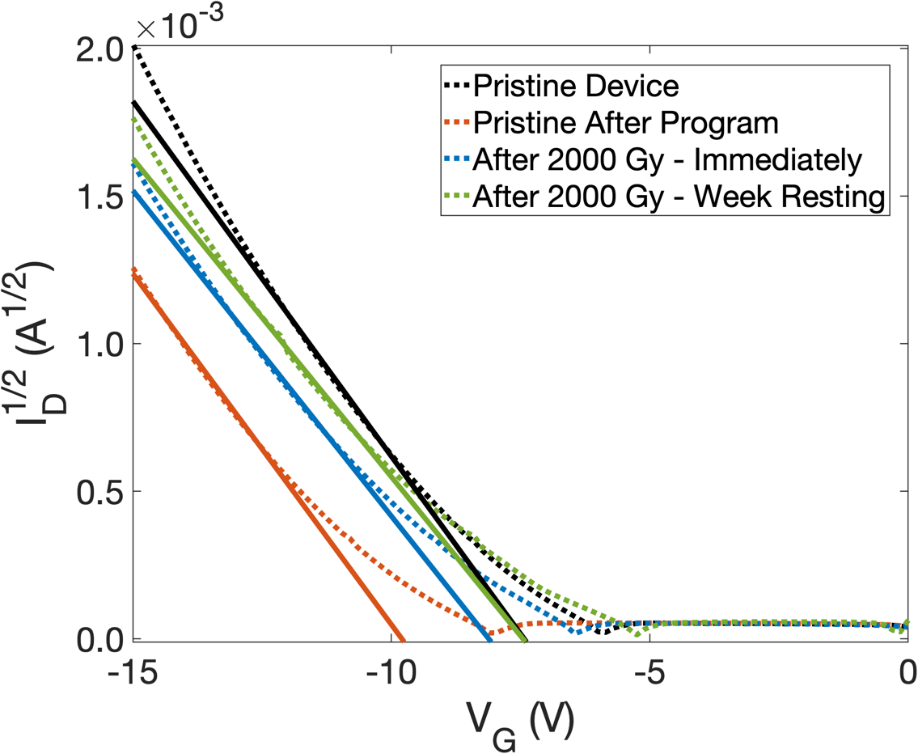


**Figure S6.** The square root of drain current as a function of gate voltage for the same device before irradiation (pristine), programmed with -80 V for 3 s, then irradiated for 2000 Gy with 6 MV FFF beam from a linear accelerator measured immediately after and measured after a week. Mobility (slope) decreases from pristine condition to after 2000 Gy, but after the device is left to rest the threshold voltage returns to the same voltage as prior to irradiation.


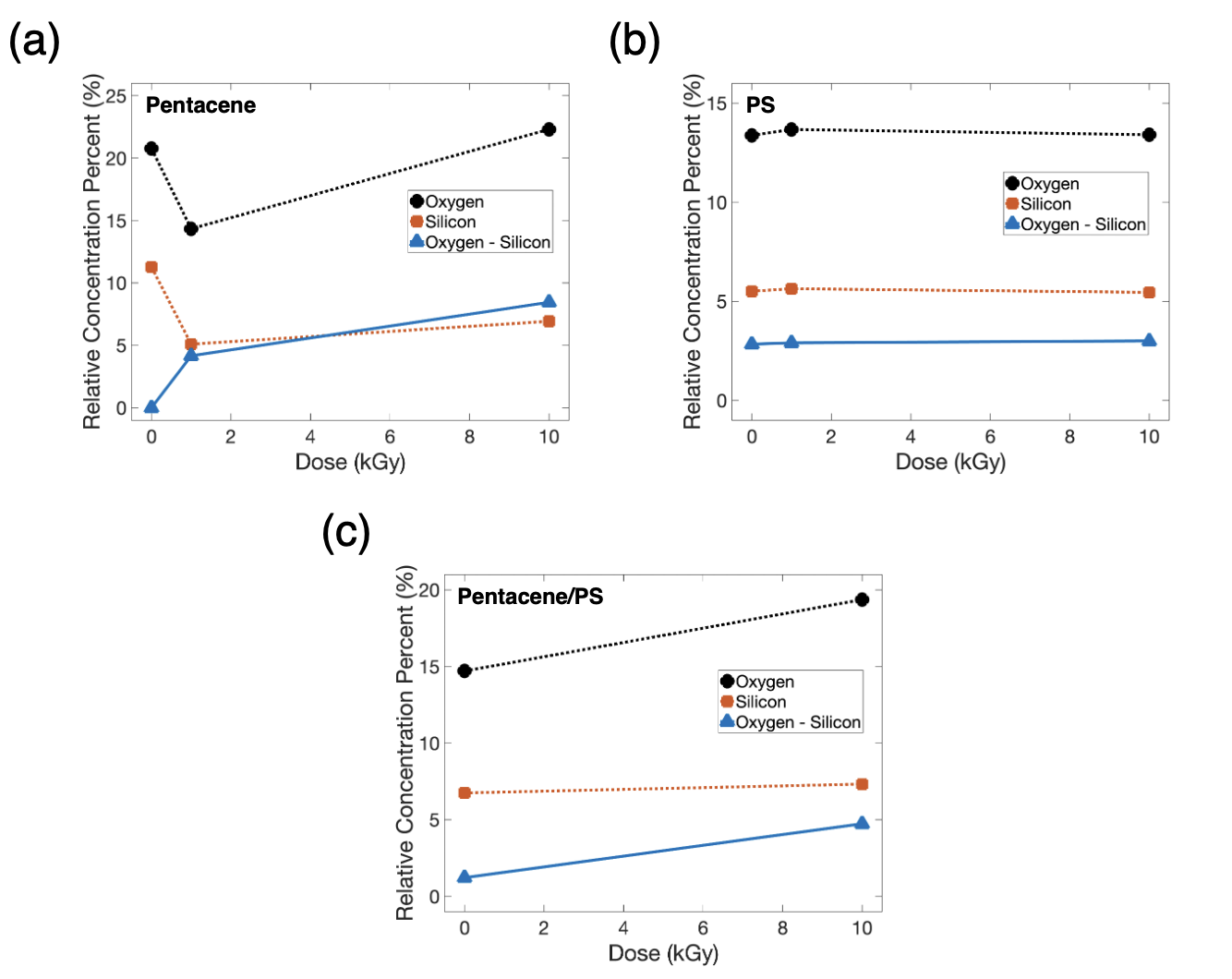


**Figure S7.** Relative concentration percentage as a function of total dose level for a) pentacene, b) polystyrene (PS), and c) pentacene/PS. Atomic concentrations were calculated from the area under the corresponding XPS core level peak and correcting for the inelastic mean free path and cross-section. Oxygen concentrations were proportional to silicon concentration and about double the value. To correct for silicon contributions, 2 times the silicon concentration was subtracted from oxygen concentration. Pentacene and pentacene/PS samples show an increase in oxygen percentage with dose and polystyrene shows no change.


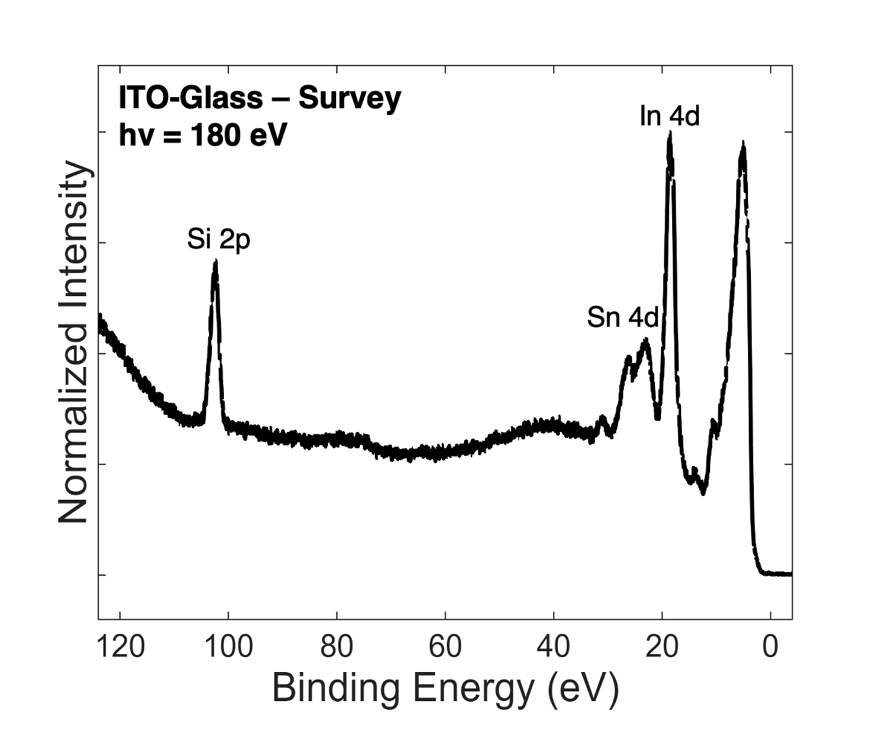


**Figure S8.** XPS survey spectrum of pristine indium-tin-oxide (ITO) coated glass substrate, demonstrating the presence of silicon on the surface of the substrates themselves.


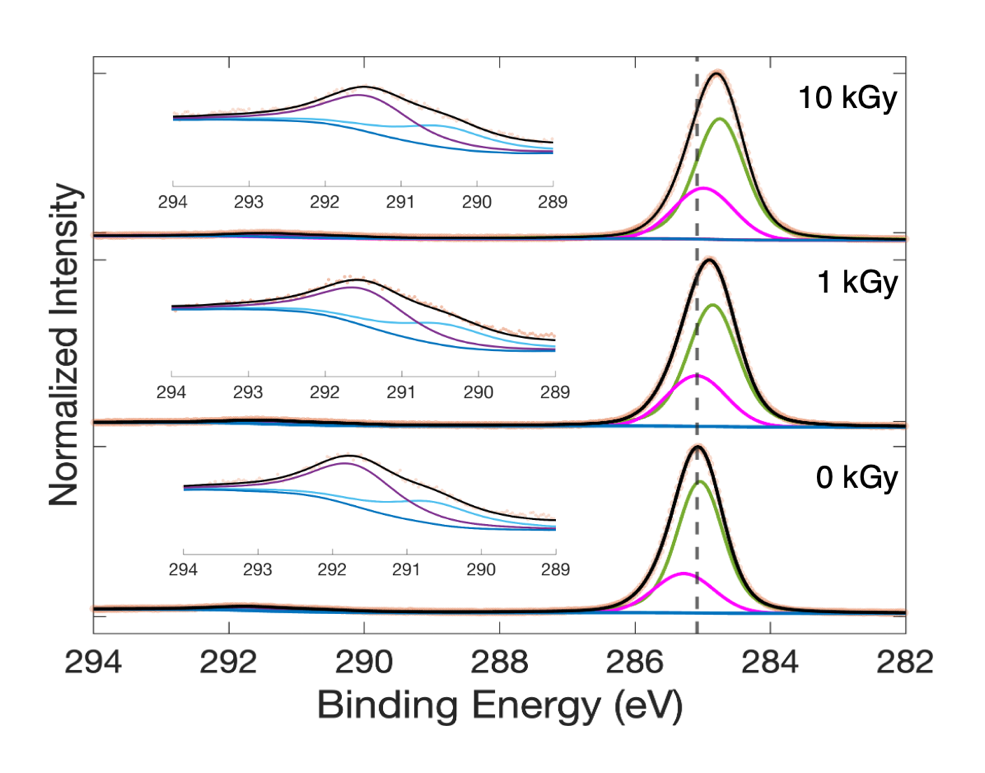


**Figure S9.** High resolution XPS spectra of pristine and irradiated single-layer polystyrene for C 1s core level (normalized to maximum). The fit components are reported for each sample.


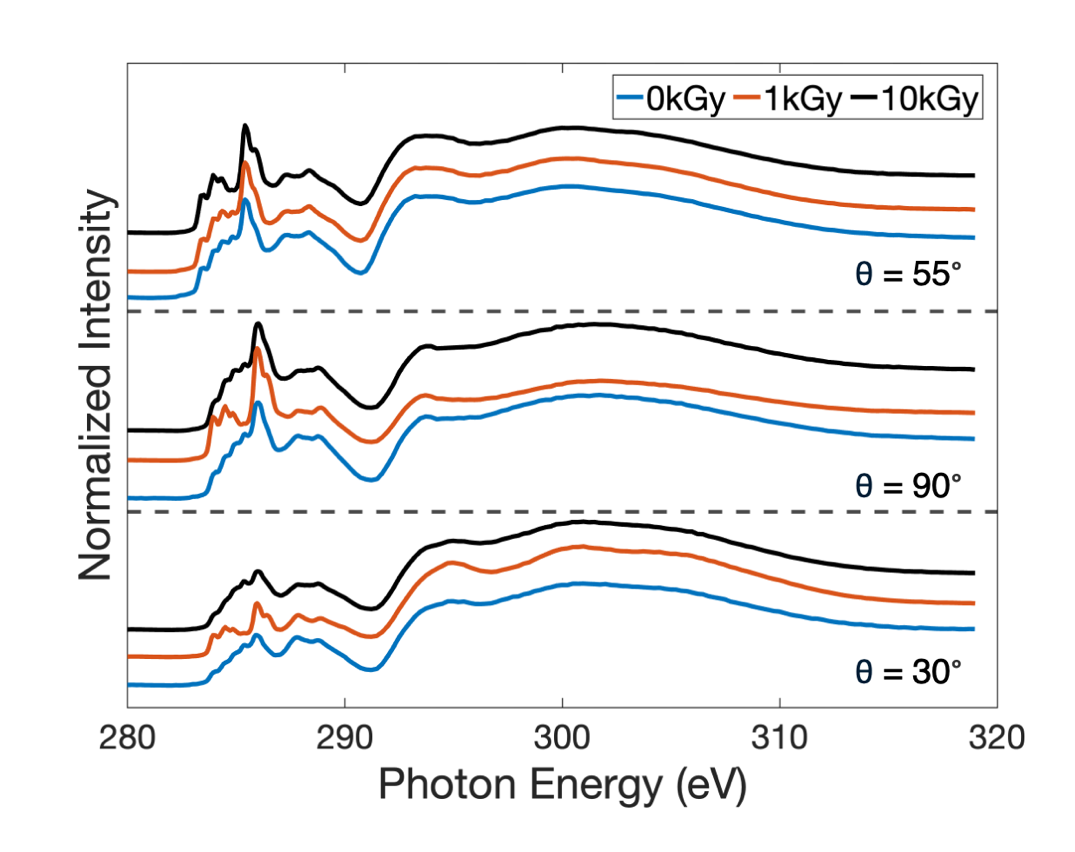


**Figure S10.** Carbon K-edge NEXAFS spectra for pristine and irradiated pentacene single-layer samples for various angles of incidence.

References

[1] L.L. Chua, J. Zaumseil, J.F. Chang, E.C.W. Ou, P.K.H. Ho, H. Sirringhaus, R.H. Friend, *Nature* **2005**, *434*, 194.
